# Distal Dorsal Thumb Mass: Giant Cell Tumor of the Tendon Sheath in an Unusual Location

**DOI:** 10.7759/cureus.41532

**Published:** 2023-07-07

**Authors:** Dieter Brummund, Angela Chang

**Affiliations:** 1 Plastic Surgery, Larkin Community Hospital, Miami, USA; 2 Anesthesiology, Aventura Hospital and Medical Center, Miami, USA

**Keywords:** fibrous histiocytoma, benign synovioma, pigmented villonodular tenosynovitis, localized nodular tenosynovitis, tenosynovial giant cell tumor

## Abstract

Giant cell tumor of the tendon sheath (GCTTS) is a common mass in the digits, hands, and upper extremities. Diagnosis is made on clinical examination, adjunctive imaging, and distinct intraoperative findings. Surgical excision is the mainstay of treatment. GCTTS are typically found on flexor surfaces with the dorsal distal thumb being an unusual location. Any surgical approach to the digit should balance oncologic margins with preserving function. GCTTS have a tendency to recur and should be approached in a methodical manner with risk factors of recurrence in mind. This case report reviews the history of GCTTS, surgical approaches to the digit, and risk factors for recurrence to achieve success in the surgical management of these tumors.

## Introduction

Masses involving the hand or digits are frequently seen by physicians. With a wide array of differential diagnoses, the exact pathology may be difficult to diagnose. The vast majority of hand tumors are benign, with the most common hand mass being a ganglion cyst.

Giant cell tumor of the tendon sheath (GCTTS), though the second most common hand mass, is rare in the population at large, with an incidence of 29 per million person-years [1]. First described in 1852 by Chassaignac as a fibrous xanthoma, a variety of names are used, including pigmented villonodular tenosynovitis, fibrous histiocytoma, and sclerosing hemangioma, reflecting competing inflammatory and neoplastic theories underlying their pathogenesis [2]. The predominant contemporary theory attributes pathogenesis to reactive synovial hyperplasia and overgrowth following an initial insult. Recent studies have implicated a somatic translocation of chromosome 1p13 leading to an overexpression of colony-stimulating factor 1 (CSF1) and an associated accumulation of macrophages, fibroblasts, and reactive cells forming the majority of the tumorous mass [3]. GCTTS is typically well-circumscribed with a pseudocapsule but may be multilobular. It frequently appears a golden or ochre color depending on the degree of hemosiderin disposition, with bands of pink collagen. Histologically, they are composed of four predominant cell types, including multinucleate giant cells, foam cells, synovial cells, and histiocyte-like cells. A classification system developed by Al-Qattan in 2001 differentiates these tumors based on whether they are contained in a single pseudocapsule (Type 1) or instead in separate nodules or satellite lesions (Type 2). Type 2 GCTTS are associated with an increased risk of recurrence attributed to incomplete excision in the index procedure [4].

The diagnosis of GCTTS is made on clinical examination and their distinct appearance intraoperatively. Transillumination plays an important role preoperatively, as GCTTS does not transilluminate. This distinguishes GCTTS from the more common ganglion cyst, which does transilluminate. Plain film radiography is routinely done prior to surgery to rule out bony involvement. Other imaging modalities can play an adjunctive role but are not routinely done. These include ultrasonography, which would reveal a homogenous hypoechoic mass, or magnetic resonance imaging with contrast, which would reveal a hypointense lesion on T1- and T2-weighted spin sequences [5].

Surgical excision is the mainstay of treatment. Wide exposure and methodical dissection under magnification assist in avoiding tumor spillage [6]. Given the high density of critical nerves, vessels, tendons, and ligaments in the hand, great care must be taken to avoid collateral damage during excision. If bony invasion is seen, cortical curettage is performed. If the functionality of the digit is compromised by large and destructive tumors, a digital amputation may be required. Adjuvant postoperative radiotherapy may also reduce the risk of recurrence but is not universally recommended [2]. Chemotherapy with tyrosine kinase inhibitors, such as imatinib, has been used in cases of recurrence or metastasis to the lungs. New novel drugs are currently in development, such as pexidartinib, a colony-stimulating factor 1 receptor (CSF1R) inhibitor targeting the reactive macrophage response associated with the development of these tumors [3,7]. In case of recurrence, marginal excision should be repeated.

## Case presentation

**Figure 1:**
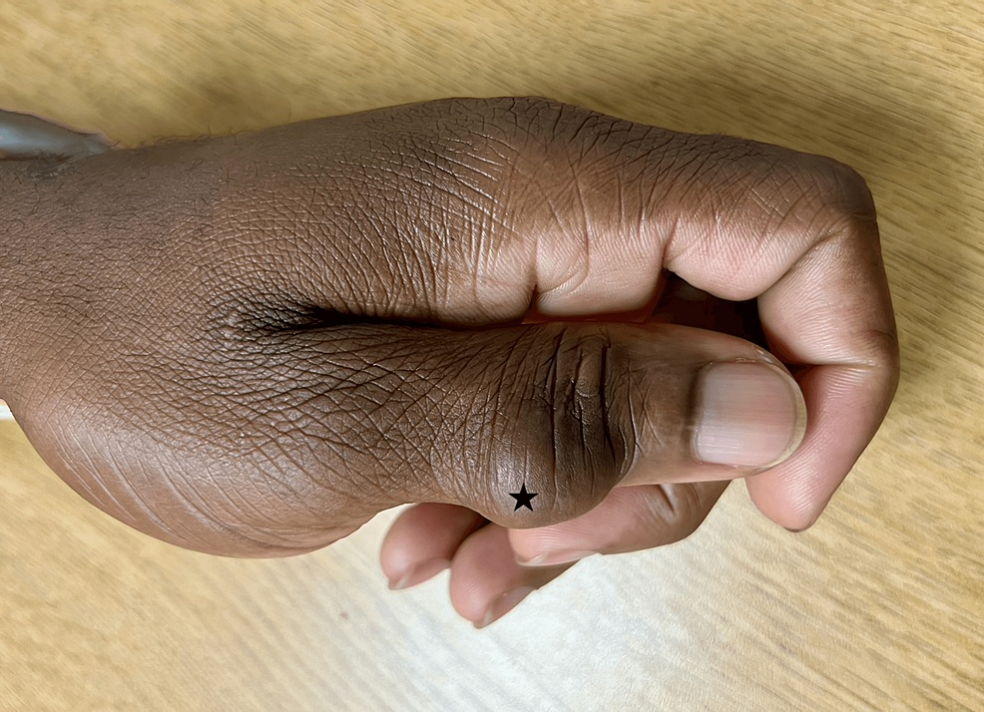
Left dorsal distal interphalangeal thumb mass (black star, dorsal view)

**Figure 2:**
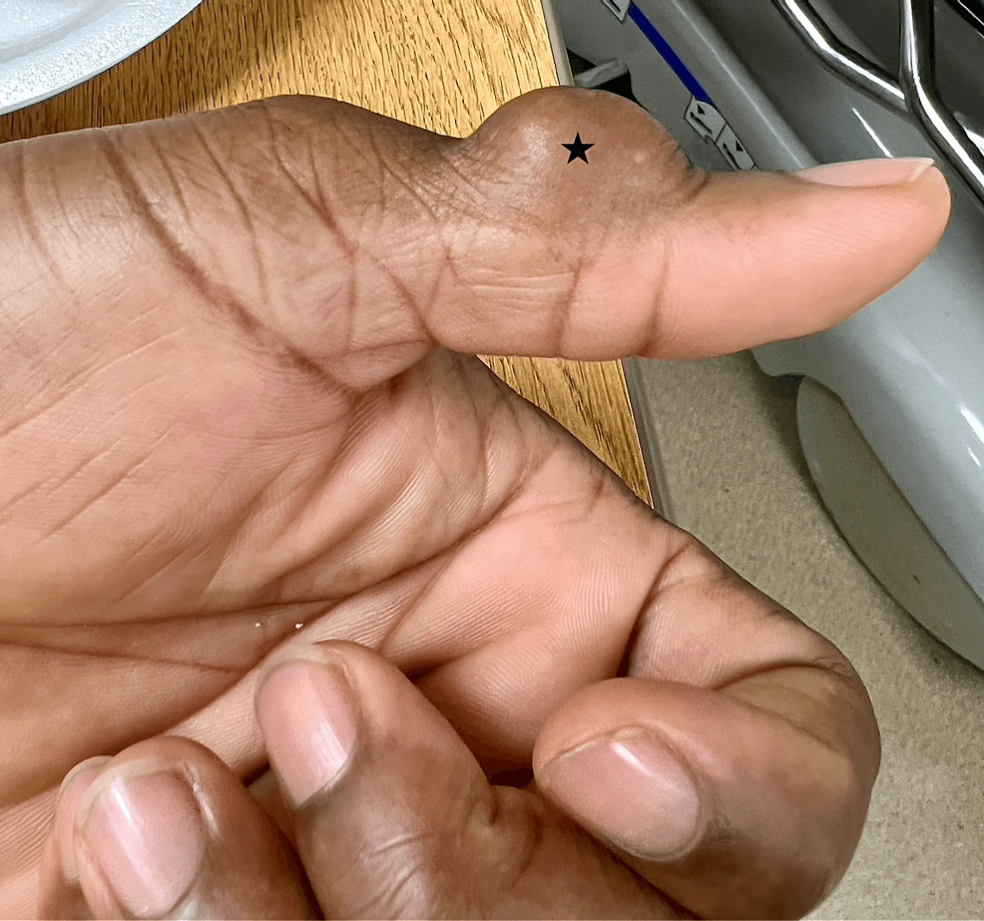
Left dorsal distal interphalangeal thumb mass (black star, lateral view)

A 38-year-old right-hand-dominant male presented with a left thumb mass overlying the dorsal distal-interphalangeal joint (Figure 1, Figure 2). The mass was not painful, but interfered with the patient's daily function due to the mass effect, leading to his decision to pursue surgical evaluation. The patient denied a history of relevant past medical illness or prior surgeries. On physical examination, the mass was firm, non-mobile, and non-tender. The affected digit was neurovascularly intact. Plain film radiography revealed no underlying boney involvement or pathology. The decision was made to proceed with surgical excision.

**Figure 3:**
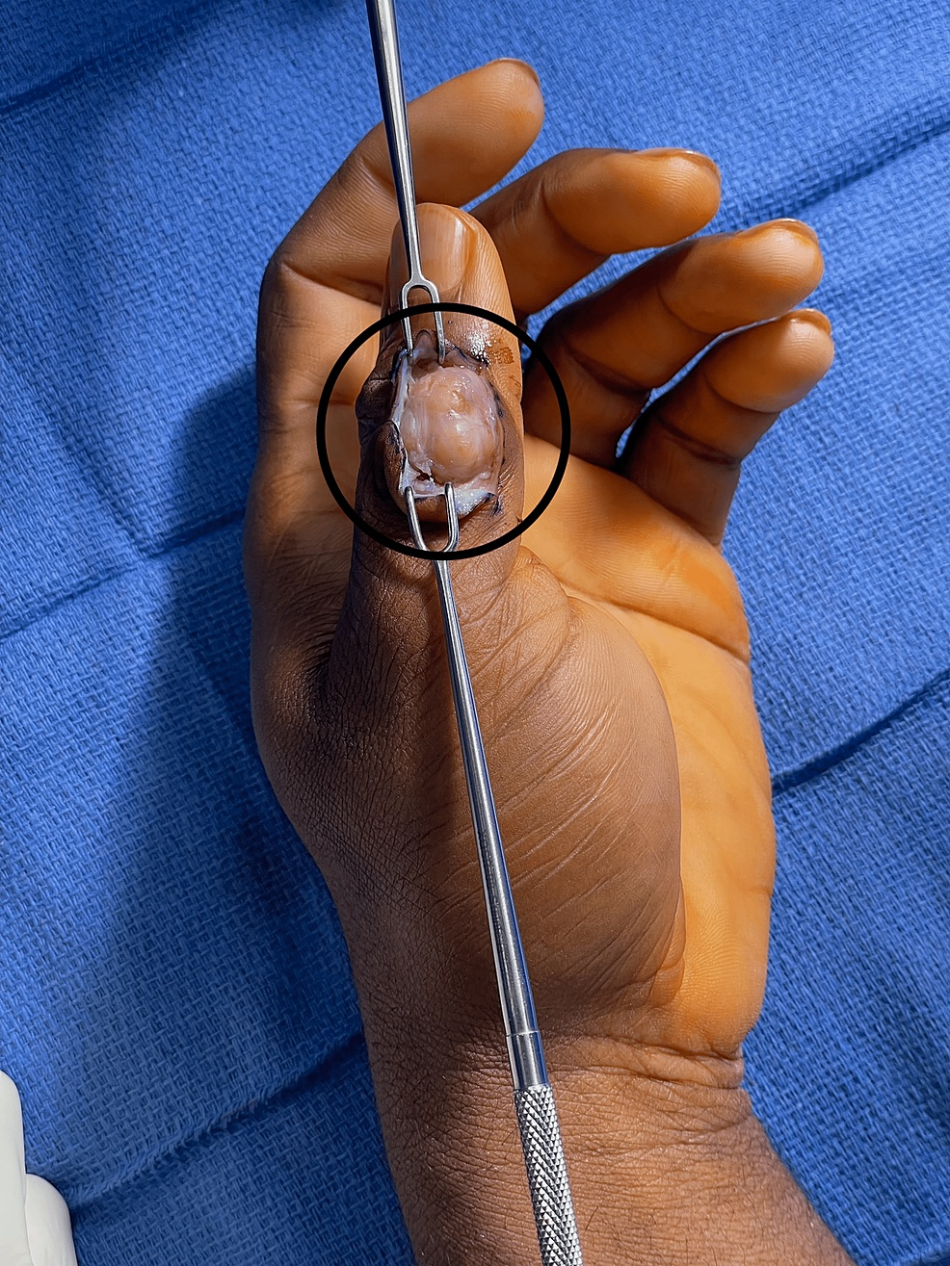
Giant cell tumor of tendon sheath in-vivo (black circle) Note the distal location radial to the distal interphalangeal joint, the large size of the mass, and the proximity and impingement upon the terminal extensor apparatus.

**Figure 4:**
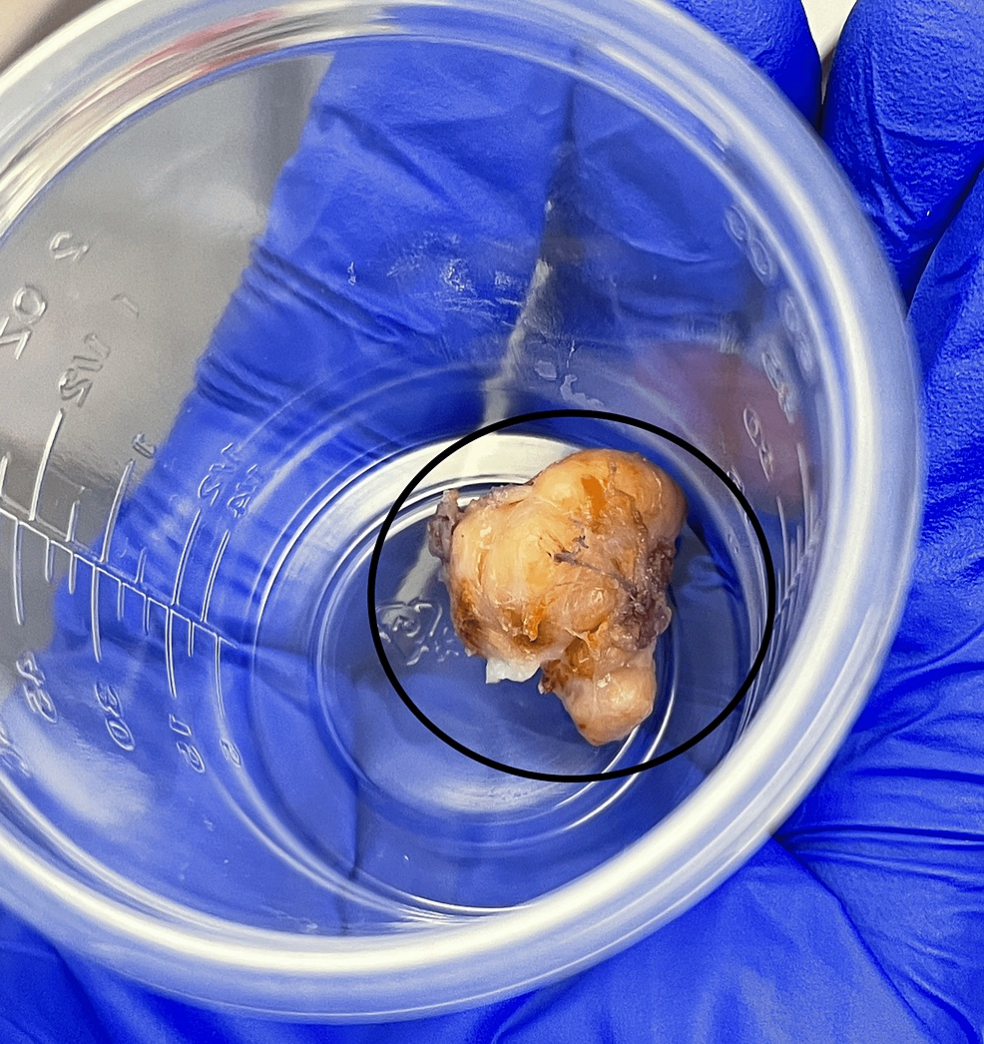
Left thumb giant cell tumor of tendon sheath ex-vivo (black oval) Note the characteristic features, including golden color, pink collagen swirls, evidence of vascularity, and multinodular form circumscribed with a pseudocapsule.

A dorsal incision was made at the radial margin of the left thumb dorsal interphalangeal joint and sharp dissection was carried down through the subcutaneous tissues to reveal a firmly adherent, multinodular, encapsulated, golden mass (Figure 3, Figure 4). Due to the size of the mass, a transverse extension of the incision was made across the dorsal interphalangeal joint to allow for wider exposure. The mass was adjacent to and had locally displaced the terminal extensor tendon, the radial triangular ligament, and the radial digital nerve. It was found that the mass had not directly invaded these structures, and thus they were able to be preserved and protected. No satellite lesions were seen. The mass was meticulously dissected from the surrounding structures under loupe magnification.

The patient tolerated the procedure well and was discharged home. Pathological analysis postoperatively revealed pigmented villonodular synovitis with clear margins. No adjuvant radiation or chemotherapy was done. The patient had no complaints or evidence of recurrence at six months of follow-up.

## Discussion

There are several points of discussion in this case, including the unusual dorsoradial location on the thumb, the approach used, and the risk factors for recurrence.

GCTTS most commonly presents in the third through fifth decades of life as a non-tender, non-mobile, firm, slow-growing mass on the volar surface of a distal phalanx in the hand. They most frequently occur on the index finger, followed by the long finger, thumb, ring finger, and small fingers, respectively. While they are most commonly found on the volar or flexor surface [8], some studies have reported a higher frequency in a dorsal location [9]. This variation may be due to the limited number of patients studied, as well as yet unknown local factors specific to their geographic location. GCTTS most commonly presents in female patients, in the dominant hand. Symptomatic patients may have paresthesias, pain, or stiffness suggestive of local invasion and involvement of the adjacent nerves, tendon, and bone. Surgical management is indicated in symptomatic patients to limit further local damage and restore function. A prospective study of 213 patients with GCTTS of the hand by Williams et al. found only 4.7% of cases to involve the dorsal distal phalanx of the thumb as seen in this case [9].

Surgical approaches to the hand and digits balance two competing interests of complete en-bloc tumor excision with avoiding postoperative contracture, or collateral damage to adjacent tendons and neurovascular structures. Longitudinal incisions are best to approach masses of unknown pathology, as they provide wide exposure, are easy to close primarily, and allow for re-excision to obtain clear oncologic margins if needed [10]. Longitudinal incisions are typically avoided over joint creases, as they tend to form thickened hypertrophic scars, which may lead to joint contracture and impair the mobility and dexterity essential to a functional hand. Important structures that must be preserved in the digits include the extensor tendon and central slip apparatus dorsally, the flexor tendons and pulleys volarly, and the terminal neurovascular structures located volar-laterally [11]. In this case, a longitudinal dorsolateral incision was made on the radial side of the thumb. Once the proximal-distal extent of the mass was determined, the dissection proceeded ulnarly with a short extension of the incision made transversely over the dorsal interphalangeal joint's relaxed skin tension line. The redundant skin overlying the joint was preserved to limit any tension overlying the joint. This transverse extension allowed for additional exposure without further longitudinal extension of the incision and the redundant skin allowing for the least possible tension to the incision to limit postoperative contracture risk and maximize mobility.

GCTTS has a high rate of recurrence from 6% to up to 15% of cases [2,3,8,9,12,13]. Risk factors for recurrence are a widely debated topic in cases of GCTTS. Various patient, tumor, and operative technique risk factors have been assessed, including age, gender, degree of tumor encapsulation, satellite lesions, joint proximity, a distal phalanx location, invasion of adjacent extensor or flexor tendon structures or joint capsule, number of mitotic figures, nm23 gene expression, use of a surgical microscope or loupe magnification, phenol treatment, and even postoperative radiotherapy. Some studies have found associations, whereas others have not substantiated these findings. A common area of agreement, however, is the association of recurrence with tumor spillage or incomplete resection at the time of the primary excision [8,9,12,13]. In this case, the distal phalanx location and proximity to the distal interphalangeal joint are both risk factors for recurrence, with tumor encapsulation and dissection with loupe magnification being favorable factors for a successful outcome. Recurrence has been found to occur late, on average more than two years after excision [12]. The six-month follow-up, in this case, may be insufficient, and we plan to continue to observe the patient over time to monitor for recurrence.

## Conclusions

Giant cell tumor of the tendon sheath is a common mass occurring in the hand but rarely involves the dorsal distal thumb. Surgical excision is the primary treatment with long-term follow-up required to monitor for recurrence. A dorsal lateral longitudinal approach allows for wide exposure to achieve clear oncologic margins while limiting the risk of postoperative contracture and damage to adjacent structures. Care must be taken during dissection to remove all tumors and avoid tumor spillage to reduce the risk of recurrence and optimize the chance of a successful outcome.

## References

[1] Mastboom MJ, Verspoor FG, Verschoor AJ (2017). Higher incidence rates than previously known in tenosynovial giant cell tumors. Acta Orthop.

[2] Adams EL, Yoder EM, Kasdan ML (2012). Giant cell tumor of the tendon sheath: experience with 65 cases. ePlasty.

[3] West RB, Rubin BP, Miller MA (2006). A landscape effect in tenosynovial giant-cell tumor from activation of CSF1 expression by a translocation in a minority of tumor cells. Proc Natl Acad Sci U S A.

[4] Al-Qattan MM (2001). Giant cell tumours of tendon sheath: classification and recurrence rate. J Hand Surg Br.

[5] Gouin F, Noailles T (2017). Localized and diffuse forms of tenosynovial giant cell tumor (formerly giant cell tumor of the tendon sheath and pigmented villonodular synovitis). Orthop Traumatol Surg Res.

[6] Cherla D, Hahn E, Datiashvilli R (2013). Meticulous surgical excision of a localized giant cell tumor of the tendon sheath. ePlasty.

[7] Benner B, Good L, Quiroga D (2020). Pexidartinib, a novel small molecule CSF-1R inhibitor in use for tenosynovial giant cell tumor: a systematic review of pre-clinical and clinical development. Drug Des Devel Ther.

[8] Ozben H, Coskun T (2019). Giant cell tumor of tendon sheath in the hand: analysis of risk factors for recurrence in 50 cases. BMC Musculoskelet Disord.

[9] Williams J, Hodari A, Janevski P, Siddiqui A (2010). Recurrence of giant cell tumors in the hand: a prospective study. J Hand Surg Am.

[10] Bruns J, Delling G, Henne-Bruns D, Hossfeld DK (2008). Biopsy of tumors of the musculoskeletal system. Dtsch Arztebl Int.

[11] Mankin HJ, Lange TA, Spanier SS (1982). The hazards of biopsy in patients with malignant primary bone and soft-tissue tumors. J Bone Joint Surg Am.

[12] Kitagawa Y, Takai S (2020). Optimal treatment for tenosynovial giant cell tumor of the hand. J Nippon Med Sch.

[13] Lancigu R, Rabarin F, Jeudy J, Saint Cast Y, Cesari B, Fouque PA, Raimbeau G (2013). Giant cell tumors of the tendon sheaths in the hand: review of 96 patients with an average follow-up of 12 years. Orthop Traumatol Surg Res.

